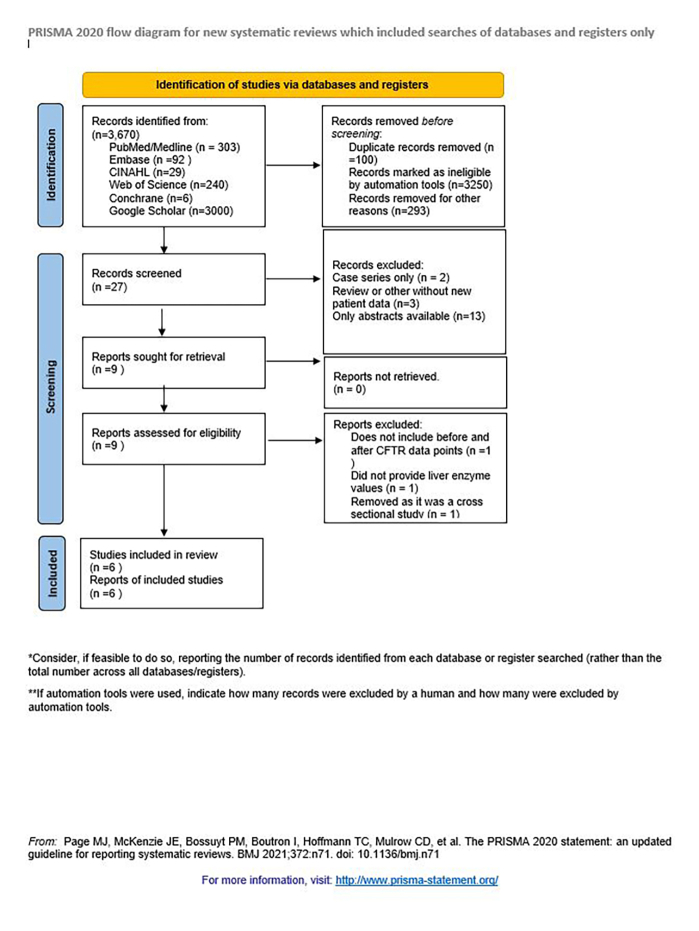# Effect of Cystic Fibrosis Transmembrane Conductance Regulator Modulators on Liver Enzymes Among Patients With Cystic Fibrosis: A Systematic Review and Meta-Analysis

**DOI:** 10.1016/j.gastha.2025.100752

**Published:** 2025-07-24

**Authors:** Tahne Vongsavath, Kyaw Min Tun, Dwaipayan Mukhopadhyay, Chun-Han Lo, Ashok Singh, Rajan Amin, Patrick Twohig, Sammy Saab, Vignan Manne

**Affiliations:** 1 Department of Internal Medicine, Kirk Kerkorian School of Medicine at UNLV, University of Nevada, Las Vegas, Nevada; 2 Division of Gastroenterology and Hepatology, Department of Medicine, Creighton University School of Medicine, Omaha, Nebraska; 3 Department of Epidemiology & Biostatistics, School of Public Health at UNLV, Las Vegas, Nevada; 4 Department of Resorts, Gaming & Golf Management, University of Nevada, Las Vegas, Nevada; 5 Department of Gastroenterology and Hepatology, Kirk Kerkorian School of Medicine at UNLV, University of Nevada, Las Vegas, Nevada; 6 Department of Gastroenterology and Hepatology, University of Rochester Medical Center, Rochester, New York; 7 Departments of Medicine and Surgery, David Geffen School of Medicine at UCLA, Los Angeles, California

**Keywords:** CFLD, CFTR modulator, CFTR safety, cystic fibrosis liver disease

## Abstract

**Backgrounds and Aims:**

This paper seeks to use existing literature to investigate the use of Cystic Fibrosis Transmembrane Conductance (CFTR) modulators and their safety and effects on the liver enzymes of cystic fibrosis (CF) patients with and without related liver disease (CFLD). This review examines the effect of CFTR modulators on liver enzymes as well as their safety profile.

**Methods:**

A comprehensive literature review across Pubmed/Medline, Embase, CINAHL, Cochrane, and Web of Science, was performed. Six articles pertaining to CFTR modulator use and liver enzyme information were included. Statistical evaluation was completed using random-effect models with a 95% confidence interval (CI), and group mean differences were analyzed with R software. Forest plots were generated for the observed effect, CI, and the weight of each study. The primary outcome was changes to serum levels of alanine transaminase (ALT), aspartate transaminase, total bilirubin, and gamma-glutamyl transferase. Secondary outcomes for this study were changes in serum biomarkers and type of CFTR modulator used. Other outcomes explored include the safety of CFTR modulator use.

**Results:**

Patients treated with lumacaftor/ivacaftor had a significant decrease in total bilirubin (−0.70 (−1.26, −0.14); *P* = .033) and gamma-glutamyl transferase (−0.98 (−1.45, −0.51); *P* = .012). While ALT had a nonsignificant trend (−0.56 (−1.54, 0.41); *P* = .20), ALT was noted to decrease significantly in CF patients (−0.90 (−1.71, −0.08); *P* = .037). When pooled there was significant change in ALT in those with and without CFLD (−0.98 (−1.45, −0.51); *P* = .012). Reported adverse events were low, with a prevalence of 38% (CI: 8.59%–80%).

**Conclusion:**

CFTR modulators may influence levels of liver enzymes in CF patients without an increased risk of adverse events.

## Introduction

Cystic fibrosis (CF) is an autosomal recessive disorder caused by mutations in the CF transmembrane conductance regulator (CFTR). Due to its role of modulation of other ion transporting channels, it can disrupt ion and water transportation leading to increased viscosity of secretions in multiple organ systems, including but not limited to the lungs, intestines, skin, pancreas, and liver.^1^^,^^2^ It is most prevalent in the Caucasian population with an estimated 75,000 people affected in North America, Europe, and Australia.^3^ However more recent epidemiologic studies have found CF to occur more frequently in non-Europeans populations and regions of the world where it had not been described before.^4^ Advances in the care of CF patients have led to earlier detection through newborn screening, formalized therapies, and prevention of malnutrition through more effective enzyme replacements and high protein diets.^4^ Due to its disruption in secretory function, overall morbidity is generally secondary to pulmonary disease. However, liver involvement in the pediatric population has been shown to increase the severity of CF by fourfold compared to those without liver involvement.^5^^,^^6^ Deemed CF-related liver disease (CFLD). The pathogenesis of CFLD is retention of toxic bile acids leading to fibrogenic and proinflammatory cytokines as well as stellate cell activation, which promotes fibrogenesis.^7^ CFLD is the third leading cause of mortality in CF patients, and an important aspect of the illness that warrants further investigation into future treatment methods. While the factors for development and progression are currently unknown, CFLD is not universal in CF patients.^8^ There is a 3:1 male predominance, with the highest incidence occurring in adolescence and rarely occurring in patients over the age of 20.^9^

Lumacaftor with ivacaftor and elexacaftor-tezacaftor-Ivacaftor are CFTR modulators approved for use in the United States that help restore the function of the defective sodium chloride ion channel^10^ While their use has shown improved pulmonary outcomes in CF patients, their effect on the liver is not currently known. Theoretically, via their effect on secretory function, they may help to increase bicarbonate secretion and decrease intestinal inflammation assisting in reduction of backflow of toxic bile products decreasing overall liver involvement.^11^ Baseline intestinal inflammation can promote hepatic inflammation by disrupting intestinal barrier function, which in turn leads to activation of hepatic stellate cells and Kupffer cells.^12^ The ability of CFTR modulators to assist in improving secretory function suggests utility in their use of CFLD. We performed a systematic review and meta-analysis to evaluate the safety and liver related effects of the different CFTR modulators on the liver enzymes of patients with CF with and without CFLD.

## Methods

### Search Strategy

We performed a comprehensive literature search across major databases (Pubmed/Medline, Embase, CINAHL, Cochrane, and Web of Science) using variations of the keywords “modulator”, “regulator”, “liver”, and “cystic fibrosis” to identify original studies published from inception through March 2, 2023. There were a total of 3670 studies available for review. Results narrowed via use of automation tools removing duplicates, and results were limited to human studies published in English. The remaining studies were then reviewed for eligibility criteria.

### Eligibility criteria

Inclusion criteria: (1) patients with CF (2) adult and pediatric patients; (3) reporting of patient data and outcomes after use of CFTR; (4) patients of any sex (5) reported follow-up; and (6) at least moderate quality of evidence.

Exclusion criteria: (1) individual case reports which reflect unique cases and significant bias; (2) published abstracts, letters to editor, and commentaries which do not require detailed patient data or an extensive review process; (3) studies without patient data; (4) non-English studies; and (5) animal studies.

### Quality assessment

Revised Cochrane risk-of-bias tool was used to evaluate the methodological quality of randomized controlled trials. It is a revised version of the original Cochrane risk-of-bias tool that has been widely used in systematic reviews. The tool consists of 5 domains: randomization process, derivations from intended interventions, missing outcome data, measurement of the outcome, and selection of the reported result. The overall risk of bias for each randomized controlled trial is determined high, low, or some concern based on the individual elements in the 5 domains.^13^

The Newcastle-Ottawa Scale was used to evaluate the methodological quality in case–control and cohort studies. Risk of bias regarding selection of subjects, comparability of subjects, and assessment of the exposure and outcome was graded by using a star system corresponding to 9 items. A study was categorized as low risk of bias if a total of 8 to 9 stars were allocated, medium risk of bias if 6 to 7 stars were allocated, and high risk of bias if the study was given ≤5 stars.^14^

In the final selection stage, only studies with at least moderate level of evidence were included. Quality appraisal was performed by at least 2 of the following authors (T.V., L.H., C.H., A.K., J.H.). If there was any disagreement, a senior reviewer (K.M.T.) evaluated the article and achieved consensus through discussion. See Table A1 for quality assessment scores for each included study.

### Study outcomes

The primary outcome for this study was changes to serum levels of alanine transaminase (ALT), aspartate transaminase (AST), total bilirubin, and gamma-glutamyl transferase (GGT) in CF patients being treated with CFTR.

Secondary outcomes for this study were changes in the above-mentioned serum biomarkers in patients with CFLD, without CFLD, and the type of CFTR modulator used. Other outcomes explored in this review include the safety profile of CFTR modulator use in terms of reported adverse events (AEs) including the development of cirrhosis and need for transplant.

### Study Selection and Data Extraction

A total of 3670 articles were retrieved on initial search. Five authors (T.V., L.H., C.H.L., A.K., J.H.) independently reviewed these titles and abstracts, after which 9 articles were deemed relevant with patient data.^15^, ^16^, ^17^, ^18^, ^19^, ^20^ Full texts were then reviewed by at least 2 of the following authors (T.V., K.M.T., L.H., C.H.L., A.K., J.H.), 3 were removed due to quality scores not meeting predetermined criteria or with significant bias, after which six (3 retrospective and 3 prospective cohort studies) remaining studies fulfilled complete eligibility criteria. Studies including adult and pediatric patient populations were included, and studies were not excluded based on follow-up time. In cases of disagreement, a senior reviewer (K.M.T.) arbitrated the final decision for inclusion. Study selection process by Preferred Reporting Items for Systematic Reviews and Meta-Analyses statement is detailed in Figure A1. Summary of included studies are shown in Table 1. Institutional review board review was not required as all data were extracted from published literature and no patient intervention was directly performed.

**Table 1:** Summary of Studies Included

Author/Year [Reference]	Location	Study design, cohort size	Male (n, %)	Mean age (y)	CFTR modulator used and time on (mo)
Schnell et al/2023^15^	Erlangen, Germany	Prospective, 20	13, 65%	24.1	ETI, 6
Levitte et al/2023^16^	CA, USA	Restrospective, 91	46, 50.5%	13.76	ETI, 26.4
Drummond et al/2022^17^	Paris, France	Retrospective, 28	13, 46.4%	14.4	Lumacaftor/ivacaftor, 12
Ramsey et al/2022^18^	Ohio, USA	Retrospective, 1060	558, 52.6%	20.55	Ivacaftor, ivacaftor/lumacaftor, ivacaftor/tezacaftor, ETI, 24.6
Gelzo et al/2021^19^	Naples, Italy	Prospective, 20	10, 50%	27	Lumacaftor/ivacaftor, 9
Al Oraimi et al/2022^20^	Muscat, Oman	Prospective, 21	11, 52.4%	10.8	Ivacaftor, 12

### Statistical Analysis

Means and standard deviations of serum biomarkers, before and after initiation of CFTR modulators were pooled using random-effect models with a 95% confidence interval (CI), and standardized group mean differences were analyzed with R software (version 4.3.0). All the statistical analysis was conducted using the software R. Raw effect size data in the form of means and standard deviations of 2 groups are pooled using standardized between-group mean differences. As studies with differing demographics, treatment techniques and regimens leading to potential differences in measures effect sizes were included, we have used random-effects modeling.^21^ For further information on statistical functions utilized, please see Data A1, A–C.

All the pooled rates of standardized mean differences as well as proportions pooling were calculated using the *metacont* function in R. A generalized logistic mixed-effect model was used to estimate the pooled effect for proportions.^21^ For graphical visualization of the observed effect, CI, and the weight of each study as well as the pooled effects, we have generated Forest plots through the *Meta* package in R.^22^

## Results

**Figure 1:**
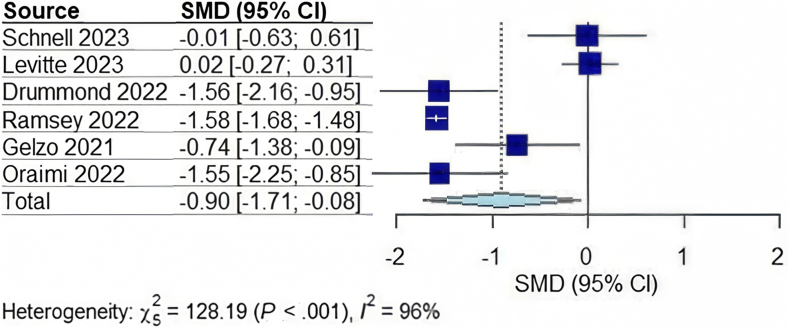
Forest plot for the change in ALT in CF patients on CF transmembrane conductance regulator modulator therapy. CI, confidence interval; SMD, standardized mean difference.

**Figure 2:**
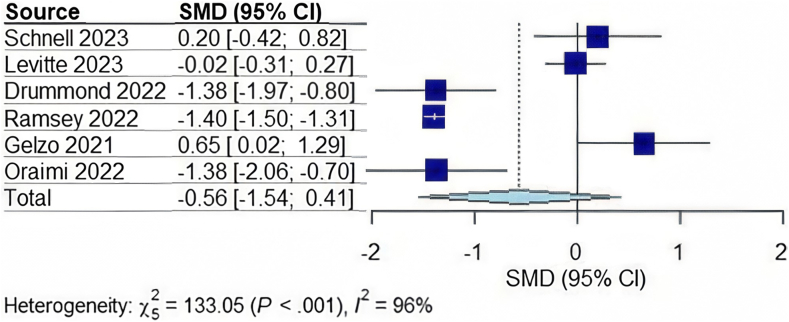
Forest plot for the change in AST in CF patients on CF transmembrane conductance regulator modulator therapy. CI, confidence interval; SMD, standardized mean difference.

**Figure 3:**
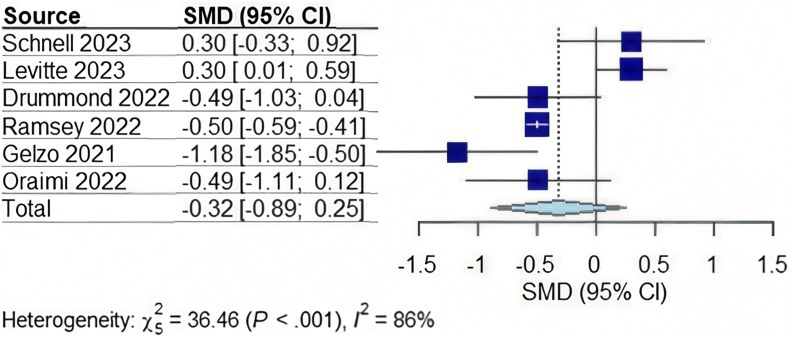
Forest plot for the change in total bilirubin in CF patients on CF transmembrane conductance regulator modulator therapy. CI, confidence interval; SMD, standardized mean difference.

In total, 1135 patients were included from the 6 studies.^11^^,^^19^^,^^20^^,^^22^, ^23^, ^24^ Two studies were completed in Europe, 3 in the USA, and 1 in Asia. The mean age of the cohort was 18.44 ± standard deviation 6.42 years with 53% of participants reported as male and average length of follow-up period was 15 months. Overall, patients treated with lumacaftor/ivacaftor did not have a significant change in AST or ALT. However, they had a significant decrease in total bilirubin and GGT as illustrated in Table 2. Patients treated with elexacaftor-tezacaftor-Ivacaftor also did not demonstrate a significant difference in AST, ALT, or GGT. See Figure 1, Figure 2, Figure 3 for the completed forest plots describing the aforementioned findings. Unlike those treated with lumacaftor/ivacaftor; however, this group also did not show a meaningful difference in total bilirubin. While some studies included small samples of patients who were concomitantly being treated with ursodiol large heterogeneity existed between each study. Only one study within this group reported a difference between platelet count before and after treatment, with an approximate 5541 platelet decrease after therapy was initiated.^16^ Differences in platelet count were not assessed in other studies in either group.

**Table 2:** Changes in Serum Biomarkers With 95% CIs in CF Patients After Starting CFTR Modulator Therapy

Change in serum biomarker	Overall change	CF patients on Lumacaftor/Ivacaftor	CF patients with CFLD	CF patients without CFLD
AST (U/L)	−0.56 (−1.54, 0.41); *P* = .20	−0.17 (−2.35, 2.01); *P* = .767	−0.87 (−6.00, 4.26); *P* = .276	−0.15 (−6.24, 5.93); *P* = .800
ALT (U/L)	−0.90 (−1.71, −0.08); *P* = .037	−0.65 (−2.43, 1.13); *P* = .256	−0.99 (−12.50, 10.50); *P* = .468	0.01 (−2.09, 2.12); *P* = .943
Total bilirubin (mg/dL)	−0.32 (−0.89,0.25); *P* = .211	−0.70 (−1.26, −0.14); *P* = .033	N/A	N/A
GGT (U/L)	−0.57 (−1.19, 0.05); *P* = .065	−0.98 (−1.45, −0.51); *P* = .012	N/A	N/A

When compared individually by presence of CFLD, patients with and without CFLD did not show significant change in AST or ALT individually; however, when pooled into one group, ALT was found to be most likely to improve significantly while receiving therapy with CFTR modulators (−0.90 [95% CI:−1.71 to −0.08; *P* = .037]). Total bilirubin and GGT levels improved in those treated with lumacaftor/ivacaftor (*P* = .033, and 0.012 respectively). Overall fibrosis scores were observed to decrease; however, pooling could not be completed as the studies used different scoring systems.

Through the studies included, the pooled prevalence of AEs reported was 38%, with CI: 8.59%–80%.^15^, ^16^, ^17^, ^18^, ^19^, ^20^ The most prevalent reported AEs was elevation in serum transaminases. One patient developed acute lymphoblastic leukemia and was removed from the study. Ramsey and colleagues also noted that patients on CFTR modulators alone had a lower incidence of development of cirrhosis or acute liver failure overall than their untreated counterparts.^18^

## Discussion

For our primary outcome, we evaluated the changes of serum enzymes in CF patients being treated with CFTR, and secondarily evaluated the changes in serum enzymes of CF patients without CFLD. When pooled, there appeared to be a statistically significant improvement in ALT. When pooled participants were treated with the Lumacaftor/Ivacaftor arm, total bilirubin and GGT also significantly improved. CFTR dysfunction causes dysregulation of innate immune pathways leading to a proinflammatory basal status in the livers of CF patients.^23^ CFTR-defective cells have increased activity of tyrosine kinases that regulate inflammatory responses as well as activate further proinflammatory signaling cascades creating a baseline proinflammatory baseline environment for these patients. It is possible that with the aid of CFTR modulators, we are able to augment the overall inflammatory response related to the inflammation seen in CFLD.^24^ When treated with Lumacaftor/Ivacaftor, total bilirubin and GGT were noted to significantly improve. As GGT and total bilirubin levels are commonly elevated in biliary pathology, it's possible that the improvement related to treatment with lumacaftor/Ivacaftor may lead to decreased biliary injury related to CF. Lumacaftor/Ivacaftor is a combination corrector and potentiator for CFTR. In the biliary system, CFTR lines the intra and hepatic ducts, thus loss of their activity may hinder biliary secretion and ability to prevent back-diffusion to toxins due to decreased barrier function to leading to elevations in GGT and total bilirubin related to biliary injury.^23^ In theory, the use of the combination therapy leads to overall increase in secretory function, thus decreasing overall biliary injury related to loss of CFTR and potentiating improvement in GGT and total bilirubin levels. As described by a study by Ramsey et al, use of CFTR modulators has been shown to be effective at reducing and delaying incidence and progression of CF cirrhosis patients in combination with ursodiol therapy compared to those receiving ursodiol alone.^18^

While the results in the pooled population showed significant change, when groups were compared individually, those with liver involvement and those without liver involvement, there was no significant change in AST or ALT. ALT changes have been noted to be a good predictor of outcome when used in groups but does not determine the course of an individual liver.^25^ It’s important to note differences in values between laboratories for reported “upper limit of normal” values. In a paper by Dufour, differences in demographics of the populace used to determine “normal”, which may lead to reporting biases especially when looking at the value of an individual or small group. This difference in individual values may also give some clarity to why when evaluated as a group, and overall trend is measured, and outliers may be overcome by the mean of the group, ALT tends to have a better predictive value.^26^

When comparing AST, because it is also formed in kidney, brain, cardiac, and skeletal muscle, optimal use of its value should be determined in relativity to ALT, which is predominant in the cytoplasmic region of hepatocytes, when discussing hepatic injury. While AST still finds utility in De Ritis ratio testing for etiology indication of hepatitis and other calculated scoring systems such as the Fibrosis-4 index, AST to platelet score, and nonalcoholic fatty liver disease Fibrosis score, its value alone should not be used for determining liver injury.^27^ Therefore, while these groups did not show significant change in liver enzymes, it is possible that the utility of ALT, on which the use of AST relies on, is more prevalent in larger participant pools. A recent study by Moiceanu et al, there were noted to be high levels of heterogeneity between the included studies, in which transaminase patterns were found to be labile and confounding. They however note some significant reduction in GGT, AP, and bilirubin possibly supporting AST and ALT as more fluctuant measures of overall liver status, and overall improvement with CFTR modulators.^28^

Overall, the safety profile of CFTR modulators in CF patients was overall well tolerated with elevation in liver serum enzymes being the most common AE. While previous studies have reported transaminitis, defined as increase in AST or ALT three times the limit of normal, such as Al Oraimi et. reporting transaminitis in 9.5% of participants, Wainwright reporting about 5.15%, and 15% in a study by McNamara.^10^^,^^20^^,^^29^^,^^30^ As liver enzyme tests are generally intermittent in the CF population and have low specificity and sensitivity, an increase in their levels may not correlate with histologic findings.^31^ While these increases may be related to initiation of treatment related to the intermittent nature of CF, the decision to decrease or stop therapy was investigator dependent and generally related to the overall level of increase. Generally, liver function returns to baseline after cessation or decreasing the dose of therapy.^32^ Therefore, it is difficult to infer the clinical significance and probable duration of these elevations. In previous studies comparing the use of Lumacaftor/Ivacaftor and a placebo arm, overall, adverse events were evenly distributed. Generally, chest discomfort or tightness has been noted more frequently when using lumacaftor monotherapy.^33^ As treatment for CF advances, and the survivability continues to prolong, the study of more individualized treatments is imminent. An improved understanding of the CFTR gene mutations will continue to allow for new developments in therapies that target specific underlying defects in CF patients and allow for mutation specific intervention. While more novel therapies that are not mutation specific are under preclinical evaluation and include gene therapy for genome editing and stem cell therapy for tissue repair.^3^

Some limitations of the studies include that the data used was via database searches, this limited the amount of pertinent data able to be viewed per study. It also caused inability to retrieve further information on each patient’s individual data, therefore we are unable to evaluate and compare other laboratory values such as type of CF mutation, and in some instances the type of CFTR modulator used in treatment. Further, as data obtained from studies using databases, case to case matching was not performed, potentially allowing for repeats of data. Some of the larger studies included only comparison arms of those on CFTR therapy or not were used, thus making the absolute efficacy of each treatment modality difficult to ascertain and leaving room for statistical error in the data used in meta-analysis. Further, because many of the included studies were via database, there may be coding errors leading to participants being inappropriately included or excluded, or unintentional reporting biases. While changes in liver markers were observed, longer duration studies are needed to follow possible development of sequela of liver disease such as portal hypertension for better understanding of clinical significance of these changes. While these limitations exist, our study highlights the significant benefit and improvement in laboratory studies such as ALT values as well as studies related to biliary injury; GGT and total bilirubin. These findings illustrate the potential for novel use of CFTR modulators for treatment of CFLD. Further, as studies unavailable in English were excluded, there is potential to have inadvertently excluded patients in geographic locations who have high numbers of database registered CF patients.^34^

## Conclusion

While further large multicenter controlled trials are needed to further validate the findings in our analysis, CFTR modulator use appears to improve the levels of liver enzymes in CF patients overall. While there are concerns for long term hepatic sequelae involved with CFTR use, improved outcomes have been observed.^18^ As enzyme values in CF patients have been noted to be somewhat labile, more studies will be needed to investigate the amount of numeric change needed to have clinical relevance in patient outcomes.^28^ Still, there is further need for comprehensive controlled trials with comparative arms of different CFTR use, CF patients of different mutation types, and to better describe and follow the long-term adverse effects related to CFTR use and liver enzyme elevations. Further, studies are needed to decipher the interplay of mild liver enzyme elevation as well as its correlation with the need to decrease or stop therapy. With the number of CF candidates for CFTR therapy rising to over 80%, the need for more potent and individualized therapy with a well described AE profile is underway.^35^

## Figures and Tables

**Supplementary Figure:**