# Supplementary material for: Effect of Cystic Fibrosis Transmembrane Conductance Regulator Modulators on Liver Enzymes Among Patients With Cystic Fibrosis: A Systematic Review and Meta-Analysis
Source: Gastro Hep Adv. 2025 Jul 24;4(9):100752. doi: 10.1016/j.gastha.2025.100752 (PMC12446518; doi:10.1016/j.gastha.2025.100752)
Supplement: Supplementary Data [file mmc1.docx]

**Supplemental Table 1** Average Quality Assessment Score**:**

| Author/Year | Title | Type of study | Type of Quality assessment used | Score |
| --- | --- | --- | --- | --- |
| Schnell 2023 [15] | Increase of liver stiffness and altered bile acid metabolism after triple CFTR modulator initiation in children and young adults with cystic fibrosis | Retrospective | NOS | 7 |
| Levitte 2023 [16] | Effects of CFTR modulators on serum biomarkers of liver fibrosis in children with cystic fibrosis | Retrospective | NOS | 7 |
| Drummond 2022 [17] | Lumacaftor-ivacaftor effects on cystic fibrosis-related liver involvement in adolescents with homozygous F508 del-CFTR | Retrospective | NOS | 5 |
| Ramsey 2022 [18] | Cystic fibrosis patients on cystic fibrosis transmembrane conductance regulator modulators have a reduced incidence of cirrhosis | Retrospective | NOS | 7 |
| Gelzo 2021 [19] | Lumacaftor/ivacaftor improves liver cholesterol metabolism but does not influence hypocholesterolemia in patients with cystic fibrosis | Prospective | NOS | 6 |
| AlOraimi 2022 [20] | Ivacaftor in Omani children with cystic fibrosis caused by p.Ser549Arg CFTR mutation | Prospective | NOS | 7 |

**Supplemental Figure 1:** Preferred Reporting Items for Systematic Reviews and Meta-Analyses (PRISMA) statement

**Supplemental Forest Plots Not included in Main Text**

1. **Forest Plot of Adverse Events**
2. **Forest Plot of overall GGT of CF patients on CFTR modulator therapy**
